# The medical treatment with pasireotide in Cushing’s disease: an Italian multicentre experience based on “real-world evidence”

**DOI:** 10.1007/s12020-018-1818-7

**Published:** 2019-04-09

**Authors:** Rosario Pivonello, Giorgio Arnaldi, Carla Scaroni, Carla Giordano, Salvo Cannavò, Davide Iacuaniello, Laura Trementino, Marialuisa Zilio, Valentina Guarnotta, Adriana Albani, Alessia Cozzolino, Grazia Michetti, Marco Boscaro, Annamaria Colao

**Affiliations:** 1 Dipartimento di Medicina Clinica e Chirurgia, Sezione di Endocrinologia, Università Federico II di Napoli, Naples, Italy; 2 Clinica di Endocrinologia e Malattie del Metabolismo, Ospedali Riuniti di Ancona, Ancona, Italy; 3 Unità Operativa di Endocrinologia, Dipartimento di Medicina, DIMED, Università di Padova, Padova, Italy; 4 Dipartimento Biomedico di Medicina Interna e Specialistica Di.Bi.MI.S, sezione di Endocrinologia, Diabetologia e Malattie Metaboliche, A.O.U.P. “Paolo Giaccone”, Palermo, Italy; 5 Dipartimento di Medicina Clinica e Sperimentale, Università di Messina, Messina, Italy; 6 Dipartimento di Medicina Sperimentale, Università “La Sapienza”, Roma, Italy

**Keywords:** Pasireotide, Cushing’s disease, Somatostatin analogues, Medical treatment, Pituitary tumour

## Abstract

A phase III study has demonstrated that 6-month pasireotide treatment induced disease control with good safety in 15–26% of patients with Cushing’s disease (CD). The aim of the current study was to evaluate the 6-month efficacy and safety of pasireotide treatment according to the real-world evidence. Thirty-two CD patients started pasireotide at the dose of 600 µg twice a day (bid) and with the chance of up-titration to 900 µg bid, or down-titration to 450 or 300 µg bid, on the basis of urinary cortisol (UC) levels or safety. Hormonal, clinical and metabolic parameters were measured at baseline and at 3-month and 6-month follow-up, whereas tumour size was evaluated at baseline and at 6-month follow-up. At baseline, 31 patients had very mild to moderate disease and 1 patient had very severe disease. Five (15.6%) patients discontinued treatment for adverse events; the remaining 27 patients (26 with very mild to moderate disease and 1 with very severe disease), reached 6-month follow-up. Considering the group of patients with very mild to moderate disease, responsiveness, defined by the normalization (<1 the upper limit of normal range, ULN) or near normalization (>1 and ≤1.1 ULN) of UC levels, was registered in 21 patients (full control in 19 and near control in 2), corresponding to 67.7% and 80.8% according to an “intention-to-treat” or “per-protocol” methodological approach, respectively. Weight, body mass index, waist circumference, as well as total and LDL-cholesterol significantly decreased, whereas fasting plasma glucose and glycated haemoglobin significantly increased. Hyperglycaemia was documented in 81.2%, whereas gastrointestinal disturbances in 40.6% of patients. In conclusion, in the real-life clinical practice, pasireotide treatment normalizes or nearly normalizes UC in at least 68% of patients with very mild to moderate disease, with consequent improvement in weight, visceral adiposity and lipid profile, despite the occurrence or deterioration of diabetes in the majority of cases, confirming the usefulness of this treatment in patients with milder disease and without uncontrolled diabetes.

## Introduction

Cushing’s disease (CD) is a rare but severe endocrine disease caused by chronic endogenous glucocorticoid excess due to a corticotroph pituitary tumour [1–4]. CD is characterized by a clinical syndrome, comprising a specific form of metabolic syndrome, including visceral obesity, hypertension, insulin resistance, glucose intolerance or diabetes mellitus (DM) and dyslipidemia, which, together with thromboembolic diathesis, contribute to determine an increased cardiovascular risk; skeletal damage, mainly characterized by osteopenia or osteoporosis, and often complicated with vertebral fractures; susceptibility to infections, which may worsen in generalized sepsis; and neuropsychiatric disorders, which may affect either cognitive or emotional function and episodically induce suicide [1–5]. This cohort of clinical manifestations and comorbidities is responsible for the increase in mortality or deterioration of quality of life [2, 4, 6–10]. Pituitary surgery is the primary therapy in the majority of patients with CD, but it is associated with initial remission rate ranging from 25 to 100% in the hands of an expert pituitary surgeon and disease relapse in up to 50% of patients [4, 11–15]. The presence of immediate persistence or early relapse, due to absent or incomplete surgical tumour resection, and late recurrences characterizing the disease, implies the need of an additional treatment in up to 75% and an average of around one-third, during the 10–20 years after surgery. Second-line treatment options include repeat pituitary surgery, pituitary radiotherapy, adrenal surgery, generally consisting in bilateral adrenalectomy, as well as medical therapy [4, 16–18]. However, the majority of historically and currently used medical treatments have not been tested in prospective randomized studies on a large population of patients with CD. The somatostatin analogue pasireotide represents a recent pituitary-directed medication used in the treatment of CD and the only medication tested in a large prospective randomized study in patients with CD [18–22]. Indeed, a recent phase III study has demonstrated that pasireotide, administered at initial dose of 600 or 900 μg twice a day (bid) subcutaneously, induced full disease control, defined on the basis of urinary cortisol (UC) levels within or below the normal range, after six months of treatment, in about 15% and 26% of patients, respectively [21]. However, these results are probably affected by the strict patient inclusion and efficacy analysis criteria characterizing this phase III study: (1) the main inclusion criterion was the demonstration of UC levels, evaluated as the average of four different urine collections, of at least 1.5 times the upper limit of the normal range (ULN), so that the study excluded great part of patients with milder disease, and actually enrolled mostly patients with moderate to severe disease, namely patients with greatly elevated UC levels, affecting the chance of a complete control of UC during treatment; (2) the primary endpoint was represented by the proportion of patients who reached a full disease control, namely normalization of UC levels at 6-month follow-up without an up-titration of pasireotide dose during the previous period of treatment, so that patients with UC normalization and disease control were considered non-responsive if the normalization of UC levels was obtained as a consequence of an up-titration of pasireotide dose; (3) the efficacy analysis was based on an “intention-to-treat” methodological approach, where patients, who discontinued the study before reaching six months of treatment, were considered non-responsive independently from the outcome on disease control during the treatment period. It is noteworthy that the requirements of this registrative clinical trial, as well as those of controlled clinical trial, are not common objectives during a real-life clinical practice, as (1) patients with milder disease are probably the patients most commonly considered for medical treatment; (2) patients are considered responsive when a full disease control, namely UC levels within or below the normal range, and, sometimes even a partial control, when UC levels are only slightly above the ULN range, is reached at the maximal permitted and/or tolerated drug dose; and (3) patients who discontinued treatment for reasons different from the inefficacy are not strictly considered as non-responsive patients, although tolerability of the drug is intrinsic part of the drug performance. The aim of the current post-marketing observational study was to evaluate the efficacy and safety of pasireotide treatment according to the real-world evidence.

## Subjects and Methods

### Patients profile

In the current post-marketing observational study, performed with routinely data collected to follow CD patients by the referral centres, 32 patients (25 females and 7 males, 21–71 years) with a diagnosis of CD started pasireotide treatment according with Italian rules for the prescription and administration of pasireotide. Twenty-five patients had one or more previous pituitary surgery; 9 patients had radiotherapy, before (1), after the first (2) or after the last (6) pituitary surgery, completed since at least three years, for stereotactic radiotherapy, or five years, for conventional radiotherapy; 2 patients had monolateral adrenalectomy (intentional monolateral adrenalectomy in 1 and unsuccessful bilateral adrenalectomy in 1) after pituitary surgery and/or radiotherapy, before entering the study and starting pasireotide treatment. Medical treatment, including cabergoline, ketoconazole, fluconazole and temozolomide, had been previously administered in 20 patients: cabergoline was used in 15 patients and ketoconazole in 11 patients (combination of cabergoline and ketoconazole in 6 patients), whereas fluconazole or temozolamide was additionally used in 1 patient. An appropriate treatment washout, at least 1 week for steroidogenesis inhibitors and at least 4 weeks for dopamine agonists, was performed before starting pasireotide treatment; temozolomide was discontinued 20 months before starting pasireotide. In summary, 4 (12.5%) patients had never received treatment (treatment-naive), 8 (25%) patients had never received medical treatment (medical treatment-naive), whereas 7 (21.9%) patients had never received surgical treatment (surgical treatment-naive) before the enrollment in the current study. The 7 surgical treatment-naive patients included 4 treatment-naive and 3 medically treated patients. The 4 treatment-naive patients did not receive pituitary surgery because of refusal (1) or negative pituitary imaging at diagnosis (3); in these last patients, an exploratory pituitary surgery was not considered a good option, whereas in the 4 patients pituitary radiotherapy and adrenal surgery were not considered an appropriate first-line therapeutic approach. The 3 medically treated patients did not receive pituitary surgery, because of refusal, preferring or accepting alternative medical treatments before starting pasireotide. At study entry, 9 (28.1%) patients had a macroadenoma, 11 (34.4%) patients had a microadenoma, and the remaining 12 (37.5%) patients had negative pituitary imaging. Among the 9 patients with macroadenoma, 2 had not been operated, 6 had been operated with tumour size reduction but persistence of residual tumour and 1 had been operated with persistence of residual tumour of the same size as before surgery. Among the 11 patients with microadenoma, 2 had not been operated, 5 had been operated with tumour size reduction but persistence of residual tumour, 1 had been operated with persistence of residual tumour of the same size as before surgery, 1 had received exploratory surgery despite a negative pituitary imaging but with subsequent evidence of microadenoma, whereas the remaining 2 had no available information on previous tumour size. Among the 12 patients with a negative pituitary imaging, 3 were not operated, 4 showed a negative pituitary imaging before and after pituitary surgery and 5 showed a microadenoma before and a negative pituitary imaging after pituitary surgery.

### Inclusion and exclusion criteria

The main inclusion criterion for patient to be enrolled into the study was the confirmation of a diagnosis of CD in active phase of disease, based on the presence of average (mean of two or three determinations performed in different days along one week) UC > ULN and/or late night salivary cortisol (LNSC) > ULN and/or increase in midnight serum cortisol (> 1.8 µg/dl), together with lack of suppression (≥ 1.8 µg/dl) in serum cortisol after low-dose (overnight 1-mg and/or 2-days 2-mg dexamethasone) suppression test and a clinical picture suggestive of CD. The diagnosis of CD needed to be confirmed by one of the following evidence: (1) histological confirmation of corticotroph pituitary tumour at a previous surgery for patients who already had surgery before starting pasireotide or (2) the presence of a pituitary macroadenoma or large (maximal diameter > 5 mm) microadenoma, or positive gradient at the inferior petrosal sinus sampling, performed anytime before starting pasireotide treatment. The exclusion criteria were represented by (1) pituitary or adrenal surgery performed less than three months before entering the study; (2) pituitary radiotherapy performed less than three years for stereotactic radiotherapy and less than five years for conventional radiotherapy before entering the study; (3) history of intolerance to somatostatin analogues; (4) risk conditions for prolonged QT syndrome; (5) uncontrolled DM, with glycated haemoglobin (HbA1c) > 9%; (6) severe liver or renal insufficiency; and (7) pregnancy.

### Patients stratification

At the time of the study enrollment, disease degree was evaluated according with baseline UC (mean of two or three determinations performed in different days along one week) and patients were classified to have very mild [ULN < 1.5, 18 (56.3%) patients], mild [ULN ≥ 1.5 and ≤ 2, 7 (21.9%) patients], moderate [ULN > 2 and ≤ 5, 6 (18.7%) patients], severe [ULN > 5 and ≤ 10, none (0%) patients] and very severe [ULN > 10, 1 (3.1%) patient] disease. The current study included the efficacy analysis, performed in the entire group of 32 patients, as well as in the subgroups, and safety analysis, performed in the entire cohort of 32 patients, and the analysis of the effect of pasireotide treatment on clinical, metabolic, hormonal and tumoral features, performed in the 27 patients who reached the 6-month follow-up. As the entire group of patients with 6-month follow-up had very mild to moderate disease with the exception of 1 patient with very severe disease, to ensure homogeneity of the results, the study cohort analysis was performed in the group of 26 patients with very mild to moderate disease and the patient with very severe disease was described separately as single case. The baseline characteristics of the 26 patients with very mild to moderate disease included in the analysis, are described in Table 1.

**Table 1:** Baseline demographic and clinical characteristics of 26 patients with very mild to moderate disease, who reached the 6-month follow-up

Characteristics	Patients no. (%)
**Females**	21 (80.8)
**Males**	5 (19.2)
**Age - years**	
Mean	47
Range	21–71
**Time since diagnosis - years**	
Mean	7.5
Range	0–17
**Previous treatment**	
Pituitary Surgery	21 (80.8)
Adrenalectomy	2 (7.7)
Medication	16 (61.5)
Pituitary irradiation	8 (30.8)
**Urinary Cortisol - ULN**	
Mean	1.55
Median	1.39
Range	0.48–2.71
**Hypercortisolism degree**	
Very mild	14 (53.8)
Mild	6 (23.1)
Moderate	6 (23.1)

### Treatment protocol

Pasireotide was administered at the initial dose of 600 µg bid subcutaneously. At 3-month follow-up, on the basis of the UC levels, pasireotide dose was adjusted according with Italian rules for prescription and administration. In particular, patients with normal UC continued the dose of 600 µg bid, whereas patients with persistently increased UC received an up-titration of the drug dose to 900 µg bid. In case of occurrence of adrenal insufficiency, diagnosed on the basis of clinical or hormonal picture, as well as in case of drug intolerance during the first period of treatment, pasireotide was reduced to 450 or 300 µg bid. During the following period of treatment, clinical and hormonal evaluation was performed every three months and pasireotide dose was adjusted on the basis of UC levels and/or clinical picture, as well as drug intolerance, according with the physician judgement, considering 300 µg bid as the minimal dose and 900 µg bid as the maximal dose. The continuation of treatment was permitted even with absent normalization of UC, if a greater than 50% decrease in UC was accompanied by clinical advantage for the patient in accordance with physician judgement.

### Efficacy analysis

The efficacy analysis included different endpoints. The primary endpoint was the disease responsiveness, in terms of normalization or significant decrease of UC levels after six months of treatment, independently from the dose of pasireotide required to achieve the disease control. In particular, patients were considered responsive when UC were ≤ ULN, or > ULN but ≤ 1.1 ULN. On this basis, responsive patients were divided in fully controlled (UC ≤ ULN, normal UC) and nearly controlled (UC > 1 and ≤ 1.1 ULN, near normal UC). The patients with UC > 1.1 ULN were considered non-responsive patients. The primary endpoint was evaluated either considering the entire cohort of 32 patients who entered the study and started pasireotide treatment (“intention-to-treat” approach) or the 27 patients who completed the six months of pasireotide treatment, with the exclusion of the 5 patients who discontinued the treatment (“per-protocol” approach). Secondary endpoints were: (1) the proportion of patients who normalized UC after three months of treatment; (2) the change in morning plasma adrenocorticotroph hormone (ACTH) and morning serum cortisol after three and six months; (3) the change in clinical and metabolic parameters after three and six months, and (4) the change in tumour size after six months of treatment.

### Safety analysis

The safety analysis included the analysis of the adverse events, occurred in the totality of 32 patients, and the interventions performed for the adverse events during the entire period of treatment. The adverse events were graded as mild, moderate or severe on the basis of the degree, according with Common Terminology Criteria for Adverse Events Version 4.0, as well as in transient or permanent, according with the presence or absence of a spontaneous remission. The change of the adverse events after the specific interventions was registered as resolution, improvement or worsening, with details of the change of single parameters, where available.

### Clinical evaluation

The clinical evaluation, performed at baseline and every three months, included the measurement of weight, body mass index (BMI), waist circumference, blood pressure and heart rate by standard methods. BMI was calculated as weight to squared height ratio. According with BMI, patients were classified in three groups: patients with normal weight (BMI < 25 kg/m^2^), overweight (BMI ≥ 25 and < 30 kg/m^2^), and obesity (BMI ≥ 30 kg/m^2^). Obesity was graded as mild (BMI ≥ 30 and < 35 kg/m^2^), moderate (BMI ≥ 35 and < 40 kg/m^2^) and severe (BMI ≥ 40 kg/m^2^) [23]. Blood pressure was recorded as mean of three values at 1–3 minutes intervals, at the right arm, in sitting posture after 5 minutes of rest, using a standard mercury sphygmomanometer. Systemic arterial hypertension was defined as systolic blood pressure (SBP) ≥ 140 mmHg and/or diastolic blood pressure (DBP) ≥ 90 mmHg, or treatment of a previously diagnosed hypertension, according with the guidelines of the American Society of Hypertension and the International Society of Hypertension [24]. Hypertension was graded as stage I (SBP ≥ 140 and < 160 mmHg or DBP ≥ 90 and < 100 mmHg), stage II (SBP ≥ 160 and < 180 mmHg or DBP ≥ 100 and < 110 mmHg), stage III (SBP ≥ 180 or DBP ≥ 110 mmHg) [24].

### Metabolic evaluation

The metabolic evaluation, performed at baseline and every three months, included the measurement of fasting plasma glucose (FPG), HbA1c, total cholesterol (Total-C), low-density lipoprotein-cholesterol (LDL-C) and high-density lipoprotein-cholesterol (HDL-C), triglycerides (TG) and liver enzymes by standard methods. At baseline, glucose tolerance was assessed by standard oral glucose tolerance test (OGTT), which was performed in the entire cohort of patients with the exception of those with a previous diagnosis of DM, whereas during the study it was assessed by the FPG and HbA1c measurement. According with the guidelines of the American Diabetes Association at baseline, impaired fasting glucose (IFG) was diagnosed if FPG was 100–125 mg/dl; impaired glucose tolerance (IGT) was diagnosed if 2-hours plasma glucose during OGTT was 140–199 mg/dl; DM was diagnosed in the presence of one of the following four criteria, in patients without previous diagnosis and/or treatment of DM: random plasma glucose ≥ 200 mg/dl in person with symptoms of hyperglycaemia, FPG ≥ 126 mg/dl, 2-hours plasma glucose during OGTT ≥ 200 mg/dl, or HbA1c ≥ 6.5% [25]. During the study, IFG was diagnosed if FPG was 100–125 mg/dl, IGT was diagnosed if HbA1c was ≥ 5.7 and < 6.5%, and DM was diagnosed if FPG ≥ 126 mg/dl and/or HbA1c ≥ 6.5%. Lipid profile was evaluated according with the guidelines of the Adult Treatment Panel-III (ATP-III): hypercholesterolaemia was defined in case of Total-C ≥ 200 mg/dl or LDL-C ≥ 130 mg/dl or HDL-C < 40 mg/dl in men and < 50 mg/dl in women, or the presence of specific treatment, whereas hypertriglyceridemia was defined in case of TG ≥ 150 mg/dl or the presence of specific treatment [26].

### Hormonal evaluation

Hypothalamic–pituitary–adrenal axis function was assessed by evaluating UC, plasma ACTH and serum cortisol levels, by commercially available kits. At the Universities of Naples and Palermo, UC as well as morning plasma ACTH and serum cortisol were measured by solid-phase chemiluminescent enzyme immunoassay. At the Ospedali Riuniti of Ancona, UC as well as morning plasma ACTH and serum cortisol, were measured by electrochemiluminescent automated assay. At the University of Messina, UC was measured by radioimmunoassay, whereas morning plasma ACTH and serum cortisol were measured by solid-phase chemiluminescent enzyme immunoassay. At the University of Padova, UC was measured by mass spectrometry, plasma ACTH by immunoradiometric assay and serum cortisol was measured by radioimmunoassay. As UC was measured with variable methodology and considering different normal ranges, these were expressed as ratio on ULN values.

### Tumoral evaluation

Pituitary tumour characteristics were studied by 1.5T magnetic resonance imaging (MRI) scanner performed at baseline and after six months in the clinical centres involved in the study. Scans were performed before and after gadolinium administration using a T1-weighted gradient recalled-echo in sagittal and coronal planes. Maximal tumour diameter was measured before and after six months of treatment when measurable.

### Statistical analysis

The statistical analysis was performed by SPSS version 21. The data are expressed as mean ± standard error (SE). The comparison between numerical variables between baseline and 3-month follow-up or 6-month follow-up was performed by pair data non-parametric tests (Wilcoxon’s test). The comparison between prevalences was performed by *χ*^2^-test, followed by Fisher’s exact test, when applicable. Significance was set at 5%.

## Results

### Patients disposition during the study

Thirty-two patients entered the study and started pasireotide treatment. Three patients discontinued pasireotide during the first three months for adverse events or accidents, whereas 2 additional patients discontinued pasireotide between three and six months of treatment because of adverse events or accidents; therefore, 29 patients reached the 3-month follow-up, whereas 27 patients reached the 6-month follow-up. Among the 27 patients starting pasireotide treatment and reaching the 6-month follow-up, 26 had very mild to moderate disease, no patients had severe disease, whereas 1 patient had very severe disease. The majority of the following sections related to the results of the study are focused of the group of 26 patients with very mild to moderate disease reaching the 6-month follow-up.

### Patients profile at baseline

The baseline profile of the 26 patients with very mild to moderate disease and 6-month follow-up included 14 (53.8%) patients with very mild, 6 (23.1 %) patients with mild and 6 (23.1%) patients with moderate disease; 3 out of 14 patients with very mild disease had normal average UC at baseline; these patients had, however, increased midnight serum cortisol and/or LNSC, together with lack of suppression at the low-dose dexamethasone suppression test and a clinical picture suggestive of disease activity, and a previous diagnosis of CD confirmed by the pathological evidence of corticotroph pituitary tumour at the previous surgery. In the group of 26 patients with very mild to moderate disease, overweight or obesity was found in 23 (88.5%) patients, with overweight in 8 (30.8%) and obesity in 15 (57.7%) patients (mild in 6, moderate in 2 and severe in 7); hypertension was found in 16 (61.5%) patients (stage I in 12 and stage II in 4); impairment of glucose metabolism was found in 14 (53.8%) patients, with IFG in 1 (3.8%), IGT in 4 (15.4%) and DM in 9 (34.6%) patients; dyslipidemia was found in 16 (61.5%) patients, with hypercholesterolaemia in 7 (26.9%), hypertriglyceridemia in 4 (15.4%) and mixed dyslipidemia in 5 (19.2%) patients. At study entry, the evidence of a pituitary tumour or tumour remnant was found in 16 (61.5%) patients, particularly a macroadenoma in 6 (23.1%) and a microadenoma in 10 (38.4%) patients, whereas a negative pituitary imaging was registered in the remaining 10 (38.5%) patients.

### Efficacy analysis

The efficacy analysis was performed considering the totality of patients entering the study and starting pasireotide treatment, and particularly the 32 patients with any degree of disease, and the 31 patients with very mild to moderate disease, which is the main focus of the current study, as well as the 28 patients with very mild to moderate disease, and increase UC at baseline, according to an “intention-to-treat” and a “per-protocol” approaches.

### Primary endpoint: responsiveness to 6-month follow-up

**Fig. 1:**
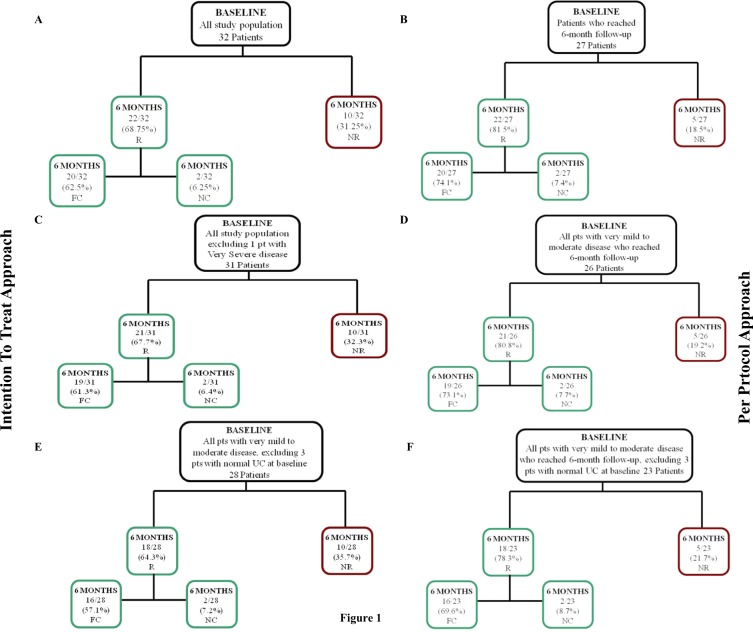
The responsiveness of patients at 6-month follow-up according to “intention-to-treat” approach and “per-protocol” approach. **a** Responsiveness of all patients (32 patients), according to “intention-to-treat” approach, dividing population in responsive (R) and non-responsive (NR), and dividing R patients in fully controlled (FC) and nearly controlled (NC). **b** Responsiveness of all patients excluding 5 patients who discontinued the treatment before 6 months (27 patients) according to “per-protocol” approach, dividing population in R and NR, and dividing R patients in FC and NC. **c** Responsiveness of all patients excluding 1 patient with very severe disease (31 patients), according to “intention-to-treat” approach, dividing population in R and NR, and dividing R patients in FC and NC. **d** Responsiveness of all patients who reached 6-month follow-up excluding 1 patient with very severe disease (26 patients), according to “per-protocol” approach, dividing population in R and NR, and dividing R patients in FC and NC. **e** Responsiveness of all patients excluding 3 patients with normal urinary cortisol (UC) at baseline (28 patients), according to “intention-to-treat” approach, dividing population in R and NR, and dividing R patients in FC and NC. **f** Responsiveness of all patients who reached 6-month follow-up excluding 3 patients with normal UC at baseline an 1 patient with very severe disease (23 patients), according to “per-protocol” approach, dividing population in R and NR, and dividing R patients in FC and NC

At 6-month follow-up, 22 patients were responsive, whereas 5 patients were non-responsive to pasireotide treatment; in particular, 20 patients were fully controlled (17 with normal UC and 3 with lower than normal UC), whereas 2 patients were nearly controlled. The remaining 5 patients discontinued the treatment before reaching the 6-month follow-up. On the basis of this evidence, considering the 32 patients with any degree of disease, according to an “intention-to-treat” approach, which refers to the totality of the patients entering the study, the proportion of responsive patients was 68.75% (22/32), whereas the proportion of non-responsive patients, including those with persistent increase of UC and those who discontinued the treatment was 31.25% (10/32). Particularly, among the responsive patients, the proportion of fully controlled was 62.5% and the proportion of nearly controlled was 6.25%, whereas the proportion of patients with persistent increase of UC was 15.625% and 15.625% was the proportion of patients who discontinued the treatment. On the other hand, according to a “per-protocol” approach, which refers to the 27 patients who reached the 6-month follow-up, the proportion of responsive patients was 81.5% (22/27), whereas the proportion of non-responsive patients, including exclusively those with persistent increase of UC, was 18.5% (5/27). Particularly, among the responsive patients, the proportion of fully controlled was 74.1% and the proportion of nearly controlled was 7.4%, whereas the proportion of patients with persistent increase of UC was 18.5%. On the other hand, considering the 31 patients with very mild to moderate disease, with the exclusion of the only patient with very severe disease, who was fully responsive to the treatment, according to an “intention-to-treat” approach, the proportion of responsive patients was 67.7% (21/31), whereas according to a “per-protocol” approach the proportion of responsive patients was 80.8% (21/26); in particular, full response was found in 61.3% and 73.1%, and partial response was obtained in 6.4% and 7.7%, for the two types of approach, respectively. Considering the 28 patients with very mild to moderate disease and baseline increased UC, with the exclusion of the 3 patients displaying normal average UC at baseline and remained responsive and fully controlled during the study, according to an “intention-to-treat” approach, the proportion of responsive patients was 64.3% (18/28), whereas according to a “per-protocol” approach the proportion of responsive patients was 78.3% (18/23); in particular, full response was found in 57.1% and 69.6%, and partial response was obtained in 7.2% and 8.7%, for the two types of approach, respectively. The responsiveness of patients to pasireotide at 6-month follow-up according to “intention-to-treat” or “per-protocol” approach is described in Fig. 1.

### Primary endpoint and disease degree

**Fig. 2:**
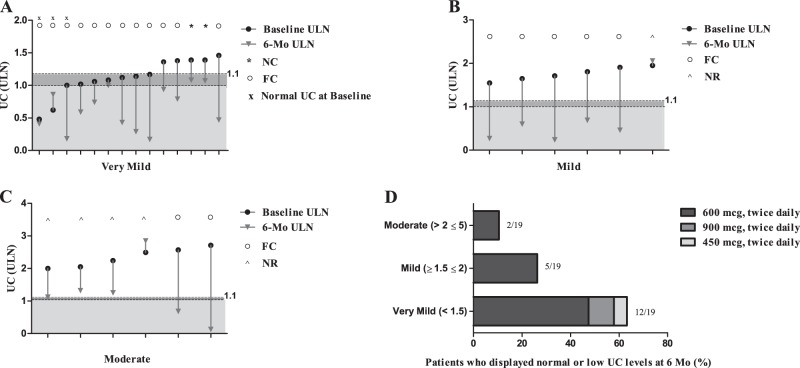
Absolute change in urinary cortisol (UC) expressed as upper limit of the normal range (ULN) from baseline to month 6 in 26 patients with very mild to moderate disease and 6-month follow-up. **a** Change in UC from baseline to month 6 in 14 patients with very mild disease, who reached 6-month follow-up divided in fully controlled (FC) and nearly controlled (NC) with indication of 3 patients with normal UC at baseline. **b** Change in UC from baseline to month 6 in 6 patients with mild disease, who reached 6-month follow-up divided in FC and non-responsive (NR). **c** Change in UC from baseline to month 6 in 6 patients with moderate disease, who reached 6-month follow-up divided in FC and NR. **d** 19 patients with very mild to moderate disease, who displayed normal or low UC at 6 months (Mo) with indication of pasireotide dose and proportion of patients divided according to disease degree

The number of responsive patients after 6 months of treatment was 14 (12 fully controlled and 2 nearly controlled) in the group with very mild, 5 fully controlled in the group with mild disease, and 2 fully controlled in the group with moderate disease; the patient with very severe disease is responsive and fully controlled at 6-month follow-up. Therefore, considering the totality of patients entering the study and starting pasireotide treatment, the percentage of responsive patients in the very mild, mild and moderate group was 77.8% (14/18), 71.4% (5/7) and 33.3% (2/6), respectively, whereas the percentage of fully controlled patients in the very mild, mild and moderate group, reaching 6-month follow-up, was 85.7% (12/14), 83.3% (5/6) and 33.3% (2/6), respectively. Indeed, the 5 patients who discontinued treatment before reaching the 6-month follow-up were distributed in the group with very mild (4, 22.2%) and in the group with mild (1, 14.3%) disease. The responsiveness and change in UC from baseline to month 6 in 26 patients with very mild to moderate disease and 6-month follow-up stratified according for the disease degree, is showed in Fig. 2.

### Primary endpoint and treatment history

Among the 4 (12.5%) treatment-naive patients, 3 (75%) were responsive, particularly 2 (50%) fully controlled and 1 (25%) nearly controlled, after 6 months of treatment, whereas 1 (25%) patient discontinued the treatment after 3 months. Among the 8 (25%) medical treatment-naive patients, 6 (75%) were responsive and fully controlled and 1 (12.5%) was non-responsive after 6 months of treatment, whereas 1 (12.5%) discontinued treatment after 3 months. Among the 7 (21.9%) surgical treatment-naive patients, 3 (42.8%) were responsive and fully controlled and 2 (28.6%) were non-responsive after 6 months of treatment, whereas 2 (28.6%) discontinued treatment, 1 after 1 month and 1 after 3 months.

### Responsiveness at 3-month follow-up

**Fig. 3:**
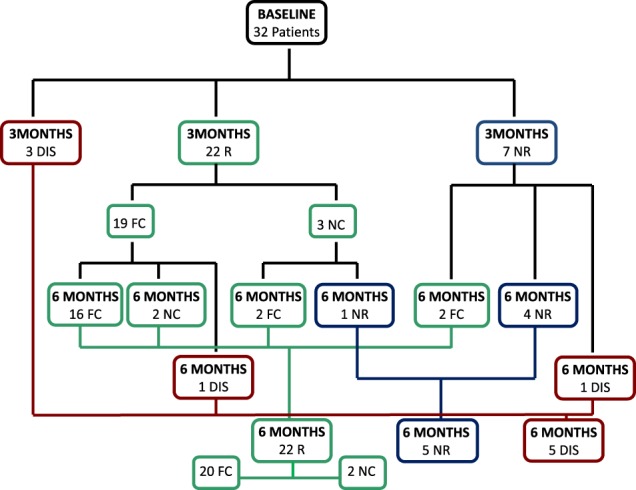
A flow-chart of patients disposition at 3-month and 6-month follow-up with classification in responsive (R), non-responsive (NR) and Discontinued (Dis). Patients R were divided in fully controlled (FC) and nearly controlled (NC)

Twenty-nine out of the 32 patients enrolled in the study reached the 3-month follow-up; 22 patients were responsive (19 fully controlled, 3 nearly controlled), whereas the remaining 7 patients were non-responsive to the treatment. Three patients discontinued the treatment before reaching the 3-month follow-up. On the basis of this evidence, according to an “intention-to-treat” approach, the proportion of responsive patients was 68.75% (59.37% fully controlled, 9.37% nearly controlled) and the proportion of non-responsive patients was 31.25% (21.87% uncontrolled and 9.37% discontinued). Conversely, according to a “per-protocol” approach, the proportion of responsive patients was 75.9% (65.5% fully controlled, 10.4% nearly controlled), whereas the proportion of non-responsive uncontrolled patients was 24.1%. The 22 responsive patients had initial very mild (14), mild (4), moderate (3) or very severe (1) disease; in particular, the 19 patients with fully controlled disease had initial very mild (13), mild (4) or moderate (2) disease, whereas the 3 patients with a nearly controlled disease had initial very mild (1), moderate (1) or very severe (1) disease. Conversely, the non-responsive patients had initial very mild (1), mild (3) and moderate (3) disease. Considering the 22 responsive patients at 3-month follow-up, 20 (90.9%) remained responsive (2 fully controlled became nearly controlled and 2 nearly controlled became fully controlled) and 1 (4.5%) became non-responsive at 6-month follow-up, whereas 1 (4.5%) discontinued treatment for bad compliance. Considering the 7 non-responsive patients at 3-month follow-up, 2 (28.6%) became responsive and fully controlled, 4 (57.1%) remained non-responsive, despite reduction of UC in 2 at 6-month follow-up, whereas 1 (14.3%) discontinued treatment because of car accident. A flow-chart of the responsiveness to the treatment at 3-month and 6-month follow-up is shown in Fig. 3.

### Effect on hormonal pattern

In the group of patients with very mild to moderate disease reaching the 6-month follow-up, a significant reduction in UC as well as in plasma ACTH was found after 3 and 6 months of treatment, whereas a significant reduction in serum cortisol was observed after 6 months of treatment (Table 2). In responsive patients, UC significantly decreased, as well as serum cortisol and plasma ACTH, after 3 and 6 months of treatment. Conversely, no significant difference in hormonal pattern between baseline and 3-month or 6-month follow-up was found in non-responsive patients, except for a significant reduction in plasma ACTH after 3 months, but not after 6 months.

**Table 2:** Changes in clinical, metabolic and hormonal parameters of 26 patients with very mild to moderate disease, who reached the 6-month follow-up

Parameter (mean ± SE)	Baseline	3 Months	*p* 3 Months	6 Months	*p* 6 Months
BMI (kg/m^2^)	34.1 ± 10.4	32.1 ± 10.4	0.000	31 ± 9.5	0.000
Weight (kg)	89.5 ± 28.5	84.5 ± 28.7	0.000	81.8 ± 27.6	0.000
Waist circumference (cm)	111.8 ± 18.8	108.9 ± 18.3	0.001	107.1 ± 18.5	0.001
Heart rate (b.p.m.)	72. ± 8.5	72.2 ± 10	NS	70 ± 8.1	NS
SBP (mm Hg)	126.7 ± 17.9	127.9 ± 20.4	NS	125.2 ± 14.2	NS
DBP (mm Hg)	81 ± 10	80.1 ± 11	NS	79.4 ± 9.5	NS
Fasting glucose (mg/dl)	94 ± 34.2	125.5 ± 47.3	0.000	122.9 ± 36.6	0.000
HbA1c (%)	5.8 ± 0.7	6.9 ± 1.2	0.000	7 ± 1.5	0.000
Total cholesterol (mg/dl)	212.5 ± 29.1	206.7 ± 25.4	NS	201.2 ± 31.5	0.035
Triglycerides (mg/dl)	137.1 ± 57.1	141.3 ± 73.1	NS	148 ± 71	NS
HDL-cholesterol (mg/dl)	56.6 ± 12.5	54.7 ± 11.7	NS	54.7 ± 11.7	NS
LDL-cholesterol (mg/dl)	129.7 ± 28.2	124.7 ± 25.1	0.045	118.1 ± 26.6	0.03
Plasma ACTH (ng/l)	83.9 ± 89.2	62.2 ± 60	0.004	57.1 ± 63.5	0.002
Serum cortisol (µg/dl)	19.6 ± 5.8	18.1 ± 6.3	NS	16.5 ± 4.9	0.01
Urinary cortisol ( µg/die)	226.2 ± 179.2	154.2 ± 158.1	0.022	144.2 ± 178.8	0.004

### Effect on clinical parameters

**Fig. 4:**
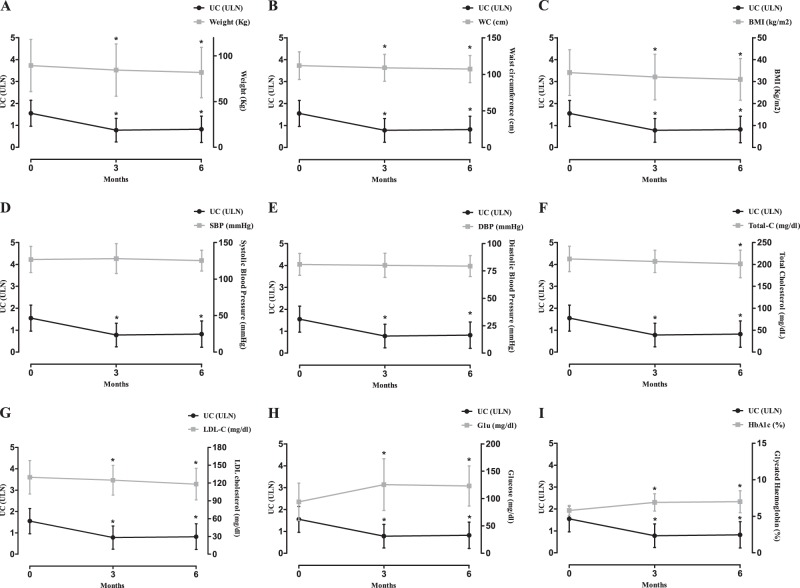
Changes in clinical and metabolic parameters compared to urinary cortisol (UC) in 26 patients with very mild to moderate disease, who reached 6-month follow-up. **a** Weight; **b** waist cirumference (WC); **c** body mass index (BMI); **d** systolic blood pression (SBP); **e** diastolic blood pression (DBP); **f** total cholesterol (total-C); **g** LDL-cholesterol (LDL-C); **h** plasma glucose (Glu); **i** glycated haemoglobin (HbA1c). *P < 0.05

In the group of patients with very mild to moderate disease reaching the 6-month follow-up, a significant reduction in weight, BMI, as well as waist circumference was found after 3 and 6 months of treatment (Table 2). Interestingly, weight and BMI were significantly decreased both in responsive and non-responsive patients, whereas waist circumference was significantly decreased in responsive, but not in non-responsive patients, after 3 and 6 months of treatment. No significant difference has been registered in the prevalence of overweight and obesity (Table 3). No significant reduction in SBP, DBP and heart rate was found after treatment (Table 2). SBP, DBP and heart rate did not significantly change either in responsive or in non-responsive patients. No significant difference has been registered in the prevalence of hypertension (Table 3). However, considering patients under antihypertensive drugs at baseline (57.7%), 20% reduced the dose or the number of antihypertensive drugs and 6.7% discontinued completely antihypertensive treatment; the totality of these patients were fully controlled. On the other hand, 13.3% of patients increased the dose of antihypertensive treatment; among these patients, 50% was uncontrolled and 50% was nearly controlled (Table 4). Changes in clinical and metabolic parameters in the group of patients with very mild to moderate disease at 6-month follow-up are shown in Fig. 4.

**Table 3:** Changes in the prevalence of comorbidities of 26 patients with very mild to moderate disease, who reached the 6-month follow-up

Comorbidity - no. (%)	Baseline	6 Months	*P*
**Overweight**	8 (30.8)	10 (38.5)	NS
**Obesity**	15 (57.7)	10 (38.5)	NS
**Arterial hypertension**	16 (61.5)	15 (57.7)	NS
**Impaired glucose metabolism**	14 (53.8)	21 (80.8)	NS
Diabetes mellitus	9 (34.6)	19 (73.1)	0.012
Impaired glucose tolerance (IGT)	4 (15.4)	1 (3.8)	NS
Impaired fasting glucose (IFG)	1 (3.8)	1 (3.8)	NS
**Dyslipidemia**	16 (61.5)	13 (50)	NS
Hypercholesterolaemia	7 (26.9)	3 (11.5)	NS
Hypertriglyceridemia	4 (15.4)	5 (19.2)	NS
Mixed dyslipidemia	5 (19.2)	5 (19.2)	NS

**Table 4:** Antihypertensive, antidiabetic, lipid-lowering treatment of 26 patients with very mild to moderate disease, who reached the 6-month follow-up

Comorbidity – no. (%)	Baseline	6 Months	Dose variation
Hypertension*	**15 treated pts**	**14 treated pts**	
1 Drug – no (%)	5 (33.3)	6 (42.8)	4 (=) 1(↑) 1(↓)
2 Drugs – no (%)	5 (33.3)	4 (28.6)	4 (=)
3 Drugs – no (%)	3 (20)	4 (28.6)	1 (=) 1(↑) 2 (↓)
≥4 Drugs – no (%)	2 (13.3)	0 (0)	1 (X)
Diabetes Mellitus**	**5 treated pts**	**16 treated pts**	
1 Drug – no (%)	4 (80)	7 (43.7)	6 (N) 1 (↑)
2 Drugs – no (%)	1 (20)	5 (31.2)	3 (↑) 2 (N)
3 Drugs – no (%)	0 (0)	4 (25)	4 (↑)
IGT/IFG**	**3 treated pts**	**1 treated pt**	
1 Drug – no (%)	3 (100)	1 (100)	1 (N)
Dyslipidemia (hypercholesterolaemia, hypertriglyceridemia, mixed dyslipidemia)***	**4 treated pts**	**4 treated pts**	
1 Drug – no (%)	3 (75)	3 (75)	2 (=) 1 (↓)
2 Drugs – no (%)	1 (25)	1 (25)	1 (=)

(=): Patients who did not change the drug dose; (↑): Patients who increased the drug dose; (↓): Patients who reduced the drug dose; (N): Patients who started the treatment; (X): Patients who stopped the treatment. *Antihypertensive drugs: Angiotensin converting enzyme-inhibitors (ACE-I), Angiotensin receptor blockers (ARBs), Diuretics, Beta-blockers, Calcium Antagonist. **Antidiabetic drugs: Metformin, Acarbose, Sulfonylureas, Dipeptidyl Peptidase-4 (DPP-4) inhibitors, Glucagon-like peptide-1 (GLP-1) agonists, Short-/Long- Acting Insulin. ***Lipid-lowering drugs: Simvastin, Fenofibrate, Polyunsaturated Fatty Acids, Ezetimibe

### Effect on metabolic parameters

In the group of patients with very mild to moderate disease reaching the 6-month follow-up, a significant reduction in Total-C was found after 6 months and in LDL-C after 3 and 6 months, whereas no significant difference was found in HDL-C and TG after treatment (Table 2). In responsive patients, LDL-C significantly decreased after 3 and 6 months, whereas Total-C significantly decreased after 6 months of treatment. In non-responsive patients, no significant difference was found in lipid profile. No significant difference has been registered in the prevalence of dyslipidemia (Table 3). Among patients under antidyslipidemic treatment at baseline (15.4%), 25% reduced the dose of one drug (Table 4). FPG and HbA1c significantly increased in total population, as well as in patients responsive and non-responsive after 3 and 6 months of treatment (Table 2). A significant increase has been registered in prevalence of DM (Table 3). Among patients with normal glucose profile at baseline (46.2%), 33.4% maintained normal glucose profile, 8.3% developed IFG, 8.3% developed IGT, whereas 50% developed DM. Among patients with IGT or IFG at baseline (19.2%), 100% developed DM. Among patients with DM at baseline (34.6%), 88.9% maintained DM, whereas 11.1% improved obtaining a normal glucose profile. Among patients under antidiabetic treatment at baseline (30.8%), 100% increased the dose or number of antidiabetic drugs, whereas among patients without antidiabetic treatment at baseline (69.2%), 34.6% started new antidiabetic therapy. Changes in metabolic parameters in the group of patients with very mild to moderate disease, who reached 6-month follow-up are shown in Fig. 4.

### Effect on tumour size

In the group of patients with very mild to moderate disease reaching the 6-month follow-up, 19 (73.1%) patients performed a pituitary MRI before and after treatment. At baseline, 5 patients had a macroadenoma, 8 patients had a microadenoma and the remaining 6 patients had a negative pituitary imaging. At 6-month follow-up, among the 5 macroadenomas, a reduction in maximal tumour diameter was documented in 2 (40%) cases (particularly, 1 became a microadenoma, with change of maximal diameter from 10 mm to 7 mm), a slight enlargement (maximal diameter increase from 23 to 25 mm) was observed in 1 (20%) case, whereas a stable tumour diameter was observed in the remaining 2 (40%) cases. The patient with slight enlargement of macroadenoma had previously undergone neurosurgery and radiotherapy, with relapse occurring after eight years, when three new surgeries, followed by temozolomide therapy for 20 months, had been performed with disease control that lasted for 17 months. Among the 8 microadenomas, a disappearance of tumour was registered in 2 (25%) cases, whereas a stable maximal tumour diameter was observed in the remaining 6 (75%) cases. A stable picture was documented in the 6 cases with negative pituitary imaging at baseline.

### Responsiveness of patients with normal baseline UC

Three patients with normal average UC at baseline were enrolled in the study and treated with pasireotide. Considering that the 3 patients maintained normal UC during the entire period of treatment, UC normalization could not represent a responsiveness criterion and the effect of pasireotide was evaluated considering clinical and metabolic parameters.

The first patient is a woman with a history of unsuccessful pituitary surgery and a negative post-surgery pituitary MRI, displaying, at study entry, UC between 0.4 and 0.6 ULN; nevertheless, CD diagnosis was performed on the basis of the evidence of loss of cortisol circadian rhythm, increased LNSC levels (1.7 µg/dl, normal range: < 0.58 µg/dl) and pathological response to low dose, overnight 1-mg and 2-days 2-mg, dexamethasone suppression tests. Furthermore, at study entry, patient showed a clinical picture characterized by overweight and DM, treated with metformin (2000 mg/die) and long-acting insulin (16 IU/bedtime). At 3-month and 6-month follow-up, the patient maintained an UC average of 0.6 and 0.4 ULN, respectively, at a stable pasireotide dose of 600 µg bid. Notably, after 6 months of treatment, patient obtained marked beneficial effects in the overall clinical picture, mainly including reduction of body weight (−4.5 kg), waist circumference (−2 cm), BMI (−1.7 kg/m^2^), still remaining in the overweight range, and DBP (−2 mm Hg), whereas pituitary MRI remained stably negative. Conversely, on the basis of the increased FPG levels, a change in antidiabetic treatment was necessary to obtain DM control: metformin (2000 mg/die), sitagliptin (100 mg/die) and long-acting insulin (20 IU/bedtime).

The second patient is a woman with a history of unsuccessful pituitary surgery and a microadenoma at post-surgery pituitary MRI, displaying, at study entry, UC between 0.5 and 0.8 ULN; nevertheless, CD diagnosis was performed on the basis of the evidence of loss of cortisol circadian rhythm, increased LNSC levels (2.21 µg/dl, normal range: < 0.58 µg/dl) and pathological response to low-dose, overnight 1-mg and 2-days 2-mg, dexamethasone suppression tests. Furthermore, at study entry, patient showed a clinical picture characterized by overweight, hypertension, treated with telmisartan (20 mg/die) and spironolactone (50 mg/die), and hypercholesterolaemia, treated with diet. At 3-month and 6-month follow-up, the patient maintained an UC average of 0.5 and 0.9 ULN, respectively, at a stable pasireotide dose of 600 µg bid. Notably, after 6 months of treatment, patient obtained marked beneficial effects in the overall clinical picture, mainly including reduction of body weight (−5 kg), waist circumference (−10 cm) and BMI (−1.7 kg/m^2^), reaching the value of normal weight, LDL-C (−24 mg/dl), still remaining in the hypercholesterolemic range, and TG (−4 mg/dl) levels. In addition, during the 6 months of pasireotide treatment, the antihypertensive therapy was reduced with telmisartan withdrawal and maintaining a stable dose of spironolactone (50 mg/die). No data on pituitary MRI at 6-month follow-up were available. Noteworthy, patient maintained a normal glucose metabolism, with no significant changes in FG and HbA1c levels.

The third patient is a woman with a history of unsuccessful pituitary surgery and a macroadenoma at post-surgery pituitary MRI, displaying, at study entry, normal UC average (1 ULN) but two of three UC determinations above the normal range (1.1 and 1.2 ULN); nevertheless, CD diagnosis was performed on the basis of the evidence of loss of cortisol circadian rhythm and pathological response to low-dose overnight 1 mg dexamethasone suppression test. Furthermore, at study entry, patient showed a clinical picture characterized by severe obesity, hypertension, treated with canrenone (100 mg/die) and irbesartan (300 mg/die), and DM, treated with metformin (2550 mg/die). At 3-month and 6-month follow-up, the patient reduced UC average to 0.7 and 0.2 ULN, respectively, at a stable pasireotide dose of 600 µg bid. Notably, after 6 months of treatment, patient obtained marked beneficial effects in the overall clinical picture, mainly including reduction of body weight (−16 kg) and BMI (−9 kg/m^2^), still remaining in the range of severe obesity, SBP (−30 mm Hg), DBP (−20 mm Hg), on a stable dose of antihypertensive drugs, and Total-C (−5 mg/dl). Noteworthy, pituitary MRI displayed pituitary tumour shrinkage, with the tumour changing from macroadenoma (maximal diameter: 10 mm) to microadenoma (maximal diameter: 7 mm). Conversely, on the basis of the increased FPG and HbA1c levels, a change in antidiabetic treatment was necessary to obtain DM control: metformin (2550 mg/die), liraglutide (1.8 mg/die) and repaglinide (3 mg/die).

### Response of the patient with very severe disease

A male previously unsuccessfully treated by pituitary surgery and medical treatment-naive had very severe disease at baseline, bearing a macroadenoma (maximal diameter of 45 mm). At study entry and before starting pasireotide, the patient was affected by overweight, hypertension, treated with nebivolol (5 mg/die) and canrenone (100 mg/die), DM, treated with short-acting (32 IU/die) and long-acting insulin (15 IU/die), and mixed dyslipidemia, treated with diet. The patient was responsive and fully controlled after 3 and 6 months of treatment with a stable pasireotide dose of 600 µg bid. At 6-month follow-up patient nearly normalized body weight (–12.3 Kg), displayed hypertension controlled with the same antihypertensive therapy but reduced antidiabetic treatment, withdrawing short-acting and long-acting insulin, as DM was controlled by the only metformin (1500 mg/die), whereas mixed dyslipidemia turned into hypertrigliceridemia, treated with diet. A stable size of macroadenoma was observed after 6 months of treatment.

### Adverse events and treatment discontinuation

The most frequent adverse event was hyperglycaemia, occurring in 26 (81.2%) patients, followed by gastrointestinal disorders, occurring in 13 (40.6%) patients; in particular, diarrhoea was registered in 12 (37.5%), abdominal pain in 5 (15.6%), nausea in 4 (12.5%) patients and meteorism in 1 (3.1%) patient. Moreover, cholelithiasis was registered in 2 (6.2%) patients. Less frequent adverse events included asthenia in 6 (18.7%) patients, confusion and irritability in 1 (3.1%) patient and articular pain in 1 (3.1%) patient. Three (9.4%) patients discontinued treatment before reaching the 3-month follow-up (2 after 25–30 days for gastrointestinal side effects, and 1 after 30 days for death due to ruptured aortic aneurysm), whereas 2 (6.2%) patients discontinued treatment 1–2 weeks after reaching the 3-month follow-up (1 for bad compliance, and 1 for car crash and following rehabilitation), therefore without reaching the 6-month follow-up.

Hyperglycaemia management goal was to obtain an HbA1c ≤ 7.5%, considering responders to antidiabetic drugs, patients who reached the HbA1c target. The first-line treatment mostly used for hyperglycaemia management was diet, in patients with mild hyperglycaemia, performed in 4/26 (15.4%) (75% responders; 25% unknown for drop-out), or metformin, starting in 9/26 (34.6%) (77.8% responders; 22.2% non-responders), or increase previous dose of metformin, adjusted in 3/26 (11.5%) (33.3% responders; 33.3% non-responders; 33.3% unknown for drop-out) patients. In patients already treated with the maximal tolerated dose of metformin at baseline and in case of severe hyperglycaemia, a different first-line approach was used: 2/26 (7.7%) were treated with glucagon-like peptide-1 (GLP-1) agonists (50% responders; 50% non-responders), 3/26 (11.5%) with insulin (33.3% responders; 66.7% non-responders), 1/26 (3.8%) with sulfonylurea (100% non-responders), whereas 4/26 (15.4%) with a combined therapy of 2/3 antidiabetic drugs (50% responders; 25% non-responders; 25% unknown for drop-out). A second-line approach was performed in a group of patients combining previous drugs with an additional drug, including acarbose in 2 patients (50% responders; 50% non-responders), GLP-1 agonists in 3 patients (66.7% non-responders; 33.3% unknown for drop-out) and dipeptidyl peptidase-4 (DPP-4) inhibitors in 1 patient (100% responders). Overall, the hyperglycaemia management goal was reached in 17 (65.4%) patients. Regarding gastrointestinal adverse events, no pharmacological therapy was started, but only diet was observed, except for 1/13 (7.7%) patient in which pasireotide dose was reduced, obtaining symptoms resolution. Cholelithiasis was treated in 1 of 2 (50%) patients with ursodeoxycholic acid, obtaining lithiasis resolution.

## Discussion

The results of the current study demonstrated that pasireotide treatment generally at the dose of 450–900 µg bid administered subcutaneously was able to induce disease remission, in terms of control of cortisol secretion, in at least 67.7% of patients with very mild to moderate disease. In particular, a full control was obtained in 61.3% and near control in 6.4% of patients. The normalization of UC levels was associated with an improvement of clinical picture, particularly weight and waist circumference, with a positive impact on visceral obesity and lipid profile. These results suggested that pasireotide might be considered an effective treatment for two-thirds of patients without severe hypercortisolism, namely patients with UC levels ≤ 5 times the upper limit of normal range.

The treatment of CD is actually based on pituitary surgery. Indeed, pituitary surgery represents the first-line treatment for a great majority of patients, but it is associated with long-term failure in around one-third of cases, due to immediate persistence (absent or incomplete tumour resection) or late recurrence (re-growth of pituitary lesion) of the disease after unsuccessful surgery [1–3, 11–15]. Repeat pituitary surgery, pituitary radiotherapy and bilateral adrenalectomy are second-line alternative therapeutic approaches for patients not cured by pituitary surgery, but are associated with unsatisfactory efficacy and/or relevant adverse effects, being hypopituitarism a common consequence of pituitary radiotherapy and adrenal insufficiency a constant consequence of successful bilateral adrenalectomy [1, 4, 16–18].

Medical therapy has recently acquired a progressively increasing role in the therapeutic algorithm of CD, due to the development of novel agents able to directly or indirectly inhibit cortisol production or action [4, 19–22]. Among these novel agents, pasireotide has been tested as possible treatment in CD. Pasireotide is a multi-receptor targeted somatostatin analogue, able to inhibit ACTH secretion through binding with the subtype-5 somatostatin receptor, strongly expressed in human corticotroph pituitary tumours [4, 27]. Pasireotide has been firstly evaluated in a multicentre open-label phase II study in patients with de novo or persistent/recurrent CD [28]. At the dose of 600 µg bid for 15 days, pasireotide induced UC decrease in 76% and UC normalization in 17% of cases [28]. The extension phase of this study conducted in 18 patients demonstrated that pasireotide was able to maintain UC normalization in 22% of cases [29]. A subsequent large randomized, double-blind phase III study (CSOM230B2305) has evaluated the efficacy of chronic treatment with pasireotide at the dose of 600 or 900 µg bid in 162 patients with de novo or persistent/recurrent CD [21]. After 6 months of treatment, 14.6% of patients, randomized to 600 μg bid, and 26.3% of patients, randomized to 900 μg bid pasireotide, and reaching the 6-month follow-up without an up-titration of the pasireotide dose, normalized UC levels [21]. The inclusion of patients who had an up-titration of pasireotide dose raised to 15.9% (600 μg bid) and 28.8% (900 μg bid) the percentage of patients who normalized UC at 6-month follow-up [21]. After 12 months, 13.4% (600 μg bid) and 25% (900 μg bid) of patients maintained normal UC levels [21]. Moreover, a higher than 50% decrease of UC levels has been achieved in 18.3% (600 μg bid) and 12.5% (900 μg bid) of patients at 6-month follow-up, and in 15.9% (600 μg bid) and 2.5% (900 μg bid) at 12-month follow-up [21]. As overall, in accordance with the criteria of the phase III study, full or partial control of the disease was obtained in 34.2% (600 μg bid) and 41.3% (900 μg bid) of patients after 6 months, and in 29.3% (600 μg) and 27.5% (900 μg) of patients after 12 months [21]. However, both after 6 and 12 months of treatment these percentages were calculated considering as non-responders all patients withdrawn from the study, in accordance to an “intention-to-treat” approach. On the basis of the results of these studies, pasireotide has obtained the official approval by European Medicines Agency (EMA) and Food and Drug Administration (FDA) for treatment of adult patients with CD who experienced a failure of pituitary surgery or are not candidates for surgery and require medical therapeutic intervention. In accordance with this indication, pasireotide has been effectively used also as pre-surgical treatment in critically ill patients with CD [30].

The results of the current study, reporting a success rate of 68.75% in the total population and 67.7% in the patients with very mild to moderate disease, seem to be in partial disagreement with the results of the phase III study [21], where the maximal success rate, including the full and partial responsive patients reached 34.2–41.3%, with the 600 and 900 μg bid, respectively. However, this apparent discrepancy may be explained by the fact that the current study is based on real-world evidence, which generally is not influenced by the strict criteria in patient inclusion and exclusion, study design and efficacy/safety analysis of the phase III studies, and, mainly, it is almost exclusively focused on patients with very mild to moderate disease.

First, the phase III registrative study included 78% of patients with moderate to very severe disease, defined by baseline UC levels higher than 2 ULN, including moderate ( > 2 ≤ 5 ULN), severe ( > 5 ≤ 10 ULN) and very severe ( > 10 ULN). Conversely, the current study was focused on patients with very mild ( < 1.5 ULN) to moderate ( > 2 ≤ 5 ULN) disease. This could partially explain the difference in the results of the two studies, confirming that the baseline UC levels are inversely associated with the percentage of UC normalization in CD [21]. Indeed, these data strongly suggest that patients with milder disease might be the ones with the highest beneficial effect from pasireotide treatment.

Second, the primary endpoint of the phase III registrative study was represented by the proportion of patients with full disease control at 6-month follow-up without an up-titration of pasireotide dose, so that patients with UC normalization and disease control were considered non-responsive if the control was obtained following an up-titration of the drug dose; this requirement is usually not applied in the evaluation of the outcome of treatment in common clinical practice. Indeed, the endpoint of the current real-world evidence study is represented by the proportion of patients with full or near control of the disease at 6-month follow-up independently from the dose of the drug necessary for this achievement. This permits to include in the calculation of success rate all patients who were uncontrolled during the first period of treatment at the initial pasireotide dose and that required an up-titration of drug dose to reach the normalization of UC after six months of treatment. The inclusion of near control is due to the evidence that in the daily clinical practice, the reduction of UC levels when approaching the normal range might be considered satisfactory, especially if associated with an important clinical benefit. It is noteworthy that, in the current study, the percentage of near control was minimal, compared to the percentage of full control.

Third, the phase III registrative study was based on an “intention-to-treat” methodological approach, which considered in the calculation of success rate, the entire number of patients starting the pasireotide treatment, and classified as non-responsive not only those who reached the 6-month follow-up without a biochemical control, but also those who discontinued the treatment independently of the reason for discontinuation, which however could be related to adverse events, compliance or pure accidental events. The overall success rate reported in the phase III registrative study was of 37.6%. The current study has evaluated the success rate either by an “intention-to-treat” methodological approach, resulting in 67.7% success rate, or with a “per-protocol” methodological approach, which considered only the patients who completed the 6-month follow-up, resulting in a success rate of 80.8% in patients with very mild to moderate disease. These data demonstrated the difference that may be obtained in the evaluation of a treatment response in dependence of the methodological approach.

The current study has two biases, which are common in a real-world evidence study. The first bias is the evidence that a very mild disease, evaluated on the basis of very mildly elevated UC levels, is affected by the variability and fluctuations of UC levels, which might occasionally reach the normal range during the treatment even in non-responsive patients. However, the real effective responsiveness to treatment in this group of patients is testified by the clinical improvement and/or reduction of tumour mass during the six months of treatment. It should be remembered that the degree of hypercortisolism by itself seems not to be an exhaustive parameter to assess the severity of the disease and many factors may influence glucocorticoid action [31, 32]. The second bias is represented by the different hormone assays used in the different centres of this multicentre study. However, in four of the five centres UC levels were measured with the same assay method, and in the current study UC levels were also evaluated as ULN with respect to the specific range of normality, minimizing the variability of the assay.

The current study confirmed that the majority of patients normalizes UC levels during the first months of treatment. Indeed, the great majority of patients who reached the 3-month follow-up normalized cortisol secretion. In particular, all patients responsive at 3-month follow-up were confirmed to be controlled at 6-month follow-up, with the exception of 1 patient who lost the disease control. On the other hand, among the 7 non-responsive patients at 3-month follow-up, 28.6% reached disease control and 28.6% improved cortisol secretion, suggesting that some patients would require longer time or higher dose of pasireotide treatment to reach the full control of the disease [33]. It is not clear whether the the loss of the near control in one patient could be considered escape to the treatment or whether this phenomenon might reflect simple UC fluctuations or deterioration of treatment compliance.

A significant shrinkage of tumour volume was found in the current real-world evidence study as much as in the phase III registrative study [21, 34] and in single-experience reports [34–37]. Shrinkage of 40% of macroadenomas and 25% of microadenomas in the current study suggests a significant beneficial effect of pasireotide treatment on the reduction of tumour volume even in patients with very mild to moderate disease.

The current study showed that the safety associated with pasireotide in this real-world evidence study on patients with mostly very mild to moderate disease is similar to the safety observed in the phase III registrative study on patients with mostly moderate to severe disease, with hyperglycaemia being the most common and diarrhoea the second most frequent adverse event. It is noteworthy that hyperglycaemia could be controlled with the use of antidiabetic treatment in nearly 65% of patients who had a deterioration of previous glucose intolerance or DM or a new onset hyperglycaemia [38].

In conclusion, the current study based on real-world evidence demonstrated that the treatment with pasireotide is able to induce hormone control in the majority of patients with very mild to moderate CD, associated with a beneficial effect on the clinical picture and comorbidities and a decrease in tumour size, although with the deterioration of glucose metabolism, which requires specific appropriate antidiabetic treatment to be prevented or controlled. Considering the high efficacy rate associated with pasireotide treatment and the increased mortality rate, mainly due to cardiovascular complications, associated with long-term untreated hypercortisolism, the treatment of milder disease with pasireotide could be taken into consideration, although balanced with the risk of pasireotide-related hyperglycaemia and development of DM, considering the presence of a baseline normal glucose tolerance as a positive permissive factor, whereas the presence of a baseline uncontrollable DM a negative limiting factor. However, the current findings should be suitably confirmed in a larger group of patients with CD.
